# Sterility of gamma-irradiated pathogens: a new mathematical formula to calculate sterilizing doses

**DOI:** 10.1093/jrr/rraa076

**Published:** 2020-09-15

**Authors:** Eve V Singleton, Shannon C David, Justin B Davies, Timothy R Hirst, James C Paton, Michael R Beard, Farhid Hemmatzadeh, Mohammed Alsharifi

**Affiliations:** Research Centre for Infectious Diseases, and Department of Molecular and Biomedical Sciences, University of Adelaide, Adelaide, SA, 5005, Australia; Research Centre for Infectious Diseases, and Department of Molecular and Biomedical Sciences, University of Adelaide, Adelaide, SA, 5005, Australia; Australian Nuclear Science and Technology Organisation, Lucas Heights, NSW, 2234, Australia; Research Centre for Infectious Diseases, and Department of Molecular and Biomedical Sciences, University of Adelaide, Adelaide, SA, 5005, Australia; Gamma Vaccines Pty Ltd, Mountbatten Park, Yarralumla, ACT, 2600, Australia; GPN Vaccines Pty Ltd, Mountbatten Park, Yarralumla, ACT, 2600, Australia; Research Centre for Infectious Diseases, and Department of Molecular and Biomedical Sciences, University of Adelaide, Adelaide, SA, 5005, Australia; GPN Vaccines Pty Ltd, Mountbatten Park, Yarralumla, ACT, 2600, Australia; Research Centre for Infectious Diseases, and Department of Molecular and Biomedical Sciences, University of Adelaide, Adelaide, SA, 5005, Australia; School of Animal and Veterinary Sciences, University of Adelaide, Roseworthy, SA, 5371, Australia; Research Centre for Infectious Diseases, and Department of Molecular and Biomedical Sciences, University of Adelaide, Adelaide, SA, 5005, Australia; Gamma Vaccines Pty Ltd, Mountbatten Park, Yarralumla, ACT, 2600, Australia; GPN Vaccines Pty Ltd, Mountbatten Park, Yarralumla, ACT, 2600, Australia

**Keywords:** gamma-irradiation, inactivation curve, sterilizing dose, sterility assurance level

## Abstract

In recent years there has been increasing advocacy for highly immunogenic gamma-irradiated vaccines, several of which are currently in clinical or pre-clinical trials. Importantly, various methods of mathematical modelling and sterility testing are employed to ensure sterility. However, these methods are designed for materials with a low bioburden, such as food and pharmaceuticals. Consequently, current methods may not be reliable or applicable to estimate the irradiation dose required to sterilize microbiological preparations for vaccine purposes, where bioburden is deliberately high. In this study we investigated the applicability of current methods to calculate the sterilizing doses for different microbes. We generated inactivation curves that demonstrate single-hit and multiple-hit kinetics under different irradiation temperatures for high-titre preparations of pathogens with different genomic structures. Our data demonstrate that inactivation of viruses such as Influenza A virus, Zika virus, Semliki Forest virus and Newcastle Disease virus show single-hit kinetics following exposure to gamma-irradiation. In contrast, rotavirus inactivation shows multiple-hit kinetics and the sterilizing dose could not be calculated using current mathematical methods. Similarly, *Streptococcus pneumoniae* demonstrates multiple-hit kinetics. These variations in killing curves reveal an important gap in current mathematical formulae to determine sterility assurance levels. Here we propose a simple method to calculate the irradiation dose required for a single log_10_ reduction in bioburden (D_10_) value and sterilizing doses, incorporating both single- and multiple-hit kinetics, and taking into account the possible existence of a resistance shoulder for some pathogens following exposure to gamma-irradiation.

## INTRODUCTION

Gamma (γ) radiation is widely used to sterilize materials in a variety of settings. It is used in the food [1], pharmaceutical [2] and medical industries [3, 4] due to the ability of γ-radiation to inactivate pathogens through nucleic acid damage, whilst leaving proteins and other structures largely intact. Consequently, γ-radiation has also been proposed as an inactivation method to generate highly immunogenic vaccines [5]. Several groups have demonstrated the superiority of γ-radiation to traditional methods of vaccine inactivation, including formalin and β-propiolactone [6, 7]. In addition, previous publications illustrated the development of highly immunogenic γ-irradiated vaccines against influenza A virus (IAV) [5, 7–9] and *Streptococcus pneumoniae* [10, 11]. Furthermore, γ-irradiated vaccines against human immunodeficiency virus (HIV) [12], and malaria [13, 14] are currently in clinical trials.

In order to ensure vaccine safety and immunogenicity, estimating the sterilizing dose (DS) under different irradiation conditions must be carefully considered. The radiosensitivity of a pathogen can be influenced by multiple factors including genome structure [15, 16], irradiation temperature [17–19], water [20] and oxygen levels [21, 22], and the presence of free-radical scavengers [23]. Importantly, resistance to γ-radiation is inversely related to genome size [15], as the chances of a single γ-ray interacting with the genome of a given pathogen is increased if the genome is larger. Accordingly, the DSs required for bacterial species are usually lower than those required for viruses [24]. In addition, it is hypothesized that viruses with more complex genomes are more radioresistant compared to viruses with simple genome structures, as a virus with a double stranded or segmented genome may require inactivation of multiple strands or segments to prevent non-damaged segments from re-assorting in a host cell. Importantly, current standard-operating procedures related to sterilization of pathogens were developed to deal with low levels of bioburden or contamination [25–27], and a dose of 50 kGy is routinely used for sterilizing pathogens that pose a biosecurity risk [28]. In this study, we investigated the effect of irradiation conditions on the irradiation dose required to sterilize highly concentrated or radioresistant pathogens, and assessed the validity of considering 50 kGy to be a widely applicable DS.

In general, DS is calculated based on the concept of a sterility assurance level (SAL). For irradiated materials, the SAL is a given probability that any single pathogen within a sample may escape inactivation following an exposure to γ-irradiation. The International Atomic Energy Agency (IAEA) recommends a SAL of 10^−6^ for products intended to come into contact with compromised tissues [25], and so this should be applied to γ-irradiated vaccines. A SAL of 10^−6^ means that there is a one in a million chance of a single infectious particle remaining following irradiation [28]. Currently, the irradiation dose required to achieve sterility at the recommended SAL of 10^−6^ (DS_SAL_) is calculated using the formula

**Fig. 1.**
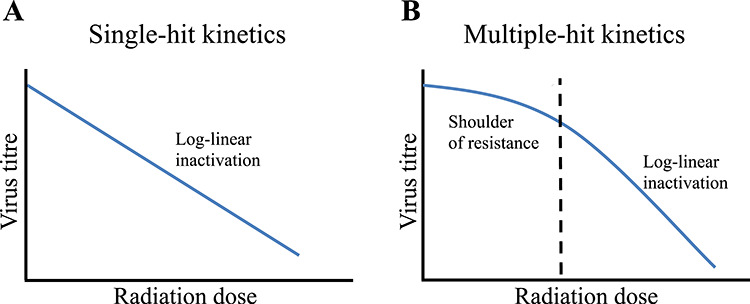
Inactivation kinetics of viruses demonstrating a model of (**A**) single-hit kinetics or (**B**) multiple-hit kinetics. Single-hit kinetics follows log–linear inactivation, whereas multiple-hit kinetics has a shoulder of resistance before damage is accumulated and log–linear inactivation occurs.

(1) \begin{equation*} {DS}_{SAL}=n\times{D}_{10} \end{equation*}

where n is the number of log_10_ reductions in bioburden required to reach a theoretical SAL of 10^−6^ and D_10_ is the irradiation dose required for a single log_10_ reduction in bioburden. Equation 1 assumes a log–linear inactivation curve, which is likely observed for viruses with simple genome structure that follow one-hit kinetics (Fig. 1A). Our recent publications, however, have shown non-linear inactivation curves (Fig. 1B) for rotavirus (RV) [29] and *S. pneumoniae* [30], demonstrating multiple-hit kinetics for complicated pathogens. While a D_10_ value is usually calculated based on the linear portion of the curve [22], ignoring the shoulder of resistance could lead to miscalculation of the irradiation dose required to achieve a SAL of 10^−6^ (or DS_SAL_).

In this study we analyse the differences in D_10_ and DS_SAL_ for pathogens with different genomic structures irradiated at different temperatures. Our data show both single-hit and multiple-hit inactivation kinetics and we have formulated a simple method to calculate the DS_SAL_. This method will ensure the shoulder of resistance is accounted for in multiple-hit inactivation models and thus allows for more accurate calculation of the SAL.

## MATERIALS AND METHODS

### Cells

Madin–Darby canine kidney (MDCK) and African green monkey kidney (Vero and MA104) cells were maintained in Dulbecco’s Modified Eagle’s Medium (DMEM) with 10% foetal bovine serum (FBS), 1% penicillin/streptomycin (P/S) and 1% 2 mM L-glutamine. For MA104 cells, 0.5% 200 mM sodium pyruvate was also added. Cells were maintained at 37°C with 5% CO_2_ in a humidified environment. Primary chicken embryo fibroblasts (CEF) were prepared from 10-day-old chicken embryos by removing the head, limbs and viscera. Bodies were fragmented then pushed through a 70 μm single cell strainer (BD). Cells were washed three times with phosphate buffer saline (PBS) by centrifugation at 1831 × *g*, then seeded into a 75cm^2^ tissue culture flask in DMEM +10% FBS and 1% P/S and kept at 37°C with 5% CO_2_ in a humidified environment. After 24 h, non-adherent cells were removed by three washes with PBS and fresh medium was added.

### Viruses

Handling of all pathogens was carried out in accordance with guidelines of the biosafety committee at the University of Adelaide and all viruses were handled inside a Class II Biosafety Cabinet. Influenza A virus (IAV) A/Puerto Rico/8/1934 (A/PR8) H1N1 and Newcastle disease virus (NDV) V4 strain were grown in the allantoic cavity of 10 day old embryonated chicken eggs (ECE). Viruses were injected at 1 × 10^3^ 50% tissue culture infectious dose (TCID_50_)/egg in PBS containing 1% P/S. Eggs were incubated at 37°C for 48 h then chilled at 4°C overnight. Infected allantoic fluid was harvested and clarified by centrifugation at 3256 × *g* at 4°C for 10 min, then stored at −80°C until required.

Semliki Forest virus (SFV) A7 strain and Zika virus (ZIKV) MRC766 (Uganda 1947) strain were grown in Vero cells and rotavirus (RV) Rh452 was grown in MA104 cells. Viruses were propagated in DMEM + 1% P/S + 1% L-Glutamine RV was additionally activated by incubation at 37°C for 1 h with 10 μg/mL TPCK-trypsin (Sigma) prior to adding to cells. Viruses were all added at an multiplicity of infection (MOI) of 0.01, and infected flasks were stored at 37°C for 24–48 h until a cytopathic effect (CPE) of ~50% of the cell monolayer was observed. Virus-containing cell culture supernatants were collected and clarified by centrifugation at 3256 × *g* at 4°C for 10 min and stored at −80°C until required.

IAV and NDV were titrated by TCID_50_ in MDCK or CEF cells, respectively, in a 96-well round-bottomed microtitre plate. Virus was activated with 0.004% trypsin then 10-fold dilutions were added to confluent cell monolayers. Plates were incubated for 3 days at 37°C with 5% CO_2_. The presence of infectious virus was determined by agglutination of 50 μL of 0.6% chicken red blood cells (cRBC) in each well. The 50% infectious dose was determined using the method described by Reed and Muench [31] and titres were given as TCID_50_/mL.

SFV and ZIKV were titrated by plaque forming assay (PFA). Confluent monolayers of Vero cells were infected with serial dilutions of virus. Adsorption of virus was allowed for 1 h then a 0.9% agar overlay was added and plates were incubated for 3 days (SFV) or 5 days (ZIKV). Cells were fixed with 5% formalin for 1 h at room temperature (RT). Overlays were removed and cells were stained with 0.2% crystal violet. Plaques were enumerated and titre was calculated as plaque-forming units (PFU)/mL.

RV was titrated by focus-forming assay (FFA) as described previously [29]. Briefly, MA104 cells were seeded in 96-well flat-bottomed microtitre plates at 6.4 × 10^3^ cells/well and plates were incubated at 37°C for 3 days until a confluent monolayer had formed. RV was activated by 10 μg/mL TPCK-trypsin for 30 min at 37°C. RV, 10-fold serially diluted, was added to wells and incubated at 37°C for 1 h to allow virus to adhere to cells. Inoculum was removed and replaced with DMEM + 1% P/S + 1% L-glutamine + 0.5% sodium pyruvate and plates were incubated for a further 18 h at 37°C. Cells were then washed, and fixed and permeabilized using acetone:methanol (1:1 ratio). RV was visualized by primary staining with a polyclonal mouse anti-RV serum for 1 h at 4°C followed by Alexa Fluor® 555 goat anti-mouse IgG (Life Technologies, USA) secondary antibody for 1 h at 4°C in the dark. Cells were also stained with 1 μg/mL DAPI (Sigma) for 10 min at RT. RV-positive cells were visualized using a Nikon Eclipse Ti fluorescent microscope and NIS-Elements AR software. Titre was calculated as focus-forming units (FFU)/mL.

### Streptococcus pneumoniae

*Streptococcus pneumoniae* strain Rx1, a capsule-deficient derivative of D39 containing two additional mutations (ΔLytA, PdT) that has been described previously [10], was used. *Streptococcus pneumoniae* was inoculated into Todd Hewitt Broth supplemented with 0.5% yeast extract (THY) medium at a starting OD_600_ of 0.02 and then grown at 37°C + 5% CO_2_ until OD reached 0.65. Bacteria were centrifuged at 4000 × *g* for 10 min at 4°C then resuspended and washed thrice in PBS. Bacteria were then resuspended in PBS + 13% glycerol at ~10^10^ colony forming unit (CFU)/mL then frozen at −80°C until required. Viable titres were measured by CFU counts on blood agar plates.

### Gamma-irradiation

Virus and bacteria stocks were shipped to the Australian Nuclear Science and Technology Organisation (ANSTO) whilst frozen on dry ice. Samples were thawed on ice or at RT, or kept frozen on dry ice as specified and were exposed to increasing doses (0–50 kGy) of γ-radiation at different conditions [RT (24–27°C), cold on ice water (4–8°C) or frozen on dry-ice]. Gamma-irradiation was performed using a ^60^Co source at the ANSTO (NSW). Radiation doses were measured using calibrated Fricke or ceric cerous dosimeters. Pathogens were then titrated to measure loss of infectivity at different radiation doses. Non-irradiated controls were treated with the same conditions (room-temperature, ice or dry ice) without exposure to γ-radiation. After irradiation all samples were stored at −80°C until required.

## RESULTS

### Inactivation curves

**Fig. 2.**
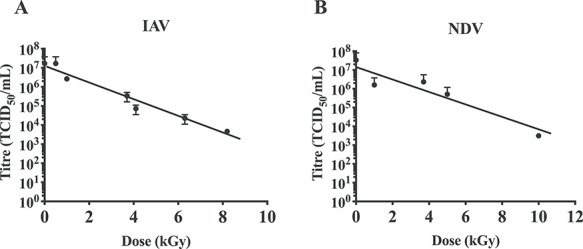
Log–linear inactivation curves of ssRNA viruses in allantoic fluid. (**A**) Influenza A virus and (**B**) Newcastle disease virus were exposed to increasing doses of γ-irradiation on dry ice. Reduced virus titre (as measured by TCID_50_/ml) for increasing irradiation doses helped to generate inactivation curves and log–linear inactivation was observed for both viruses. Data are expressed as mean ± SEM (*n* = 2). Horizontal dashed line represents background binding of virus to RBCs in the absence of a cell monolayer.

Different pathogens were exposed to incremental doses of γ-radiation and titres at each radiation dose were determined. IAV and NDV were both grown in 10-day-old ECEs and they are expected to have the same medium composition. This enabled a comparison between radiation-sensitivity of a non-segmented single-stranded RNA genome (ssRNA) genome (NDV) and a segmented ssRNA genome (IAV). Our data demonstrate log–linear inactivation for both viruses (Fig. 2), indicating single-hit inactivation kinetics.

**Fig. 3.**
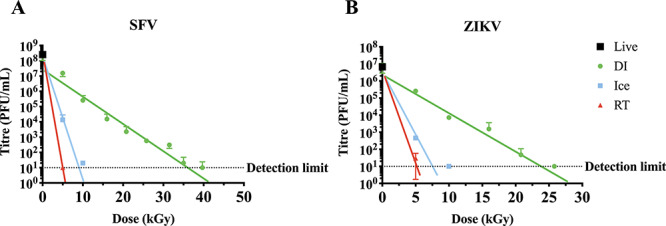
Log–linear inactivation of ssRNA viruses at different irradiation temperatures. (**A**) Semliki Forest virus and (**B**) Zika virus were exposed to increased doses of γ-irradiation on dry ice (DI) (green circles), ice (blue squares) or at room temperature (RT) (red triangles). The reduction in virus titre was estimated using plaque assay and inactivation curves were generated. Log–linear inactivation was observed for all three temperature conditions. Non-irradiated live virus was used as the starting point. Data are presented as mean ± SEM (*n* = 3). Horizontal dashed line represents detection limit.

Next, we compared the inactivation curves of SFV and ZIKV under different irradiation temperatures. Both viruses have ssRNA genomes of a comparable size, and were both grown in Vero cells using DMEM with similar medium composition. Both viruses demonstrated single-hit inactivation kinetics, with increased radiosensitivity at higher temperatures, as expected (Fig. 3).

**Fig. 4.**
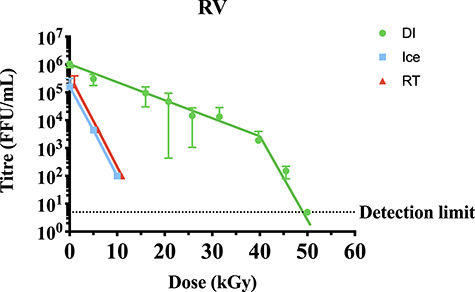
Inactivation curve of RV at different irradiation temperatures. RV was exposed to increasing doses of γ-radiation on dry ice (DI) (green circles), ice (blue squares) or at room temperature (RT) (red circles). Titre was measured by focus forming units. In contrast to both ice and RT, irradiation on DI shows an inactivation curve with multiple-hit kinetics. A shoulder of resistance appears to require an irradiation dose of 40 kGy. Data are presented as mean ± SEM (*n* = 2).

We then analysed the inactivation curve of RV, a more complex virus with a segmented and double-stranded RNA genome (dsRNA) genome structure. We have previously reported that the inactivation curve for dry ice-irradiated RV is non-linear and confirmed that here using a different strain of RV (Fig. 4). The curve shows two distinct regions. A large shoulder of resistance is observed initially, with an ~2 log loss of titre occurring between 0 to 40 kGy. After this point, a rapid decline in viable titre was observed with increased radiation dose. Importantly, calculating the DS using this inactivation curve would not be possible using current mathematical models (equation 1). Interestingly, we did not detect the multiple-hit inactivation curve for RV materials irradiated on ice or at RT (Fig. 4). This could indicate that indirect damage caused by free radicals following irradiation at higher temperatures may counteract the radioresistance of pathogens with more complex genomes.

**Table 1:** Inactivation formulae and sterility assurance levels of NDV and IAV

Virus	Formula^a^	Starting titre (TCID_50_/mL)	D_10_ (kGy)	DS (kGy)
IAV	*y* = 2 × 10^7^ × e^−1.097*x*^	1.69 × 10^7^	2.1 ± 0.16	27.77
NDV	*y* = 2 × 10^7^ × e^−0.823*x*^	3.41 × 10^7^	2.8 ± 0.53	37.86

^a^Units for *x* are kGy.

**Table 2:** Inactivation formulae and sterility assurance levels of ZIKV, SFV and RV

Virus	Irradiation condition^a^	Formula^b^	Starting titre^c^	D_10_ (kGy)	DS (kGy)
SFV	DI	*y* = 5 × 10^7^ × e^−0.418*x*^	2.55 × 10^8^	5.5 ± 0.43	79.36
	Ice	*y* = 3 × 10^8^ × e^−1.968*x*^		1.2 ± 0.23	16.86
	RT	*y* = 3 × 10^8^ × e^−3.871*x*^		<1	14.41
ZIKV	DI	*y* = 7 × 10^6^ × e^−0.625*x*^	6.75 × 10^6^	4.2 ± 0.35	54.10
	Ice	*y* = 9 × 10^6^ × e^−1.986*x*^		1.2 ± 0.06	14.87
	RT	*y* = 9 × 10^6^ × e^−2.533*x*^		0.9 ± 0.31	11.66
RV	Ice	*y* = 1 × 10^5^ × e^−0.506*x*^	1.05 × 10^6^	4.6 ± 1.1	54.71
	RT	*y* = 1 × 10^5^ × e^−0.521*x*^		4.4 ± 0.02	53.13

^a^DI = Dry ice, RT = room temperature.

^b^Units for *x* are kGy.

^c^Virus titre was measured as PFU/mL for SFV and ZIKV, and FFU/mL for RV.

### Calculating sterilizing doses

For viruses demonstrating single-hit kinetics, exponential lines of best fit could be determined using the equation:

(2) \begin{equation*} y={ae}^{- bx} \end{equation*}

where *y* is the titre at a given radiation dose *x*, *a* is the starting titre, and *b* is a constant that is determined experimentally for each individual virus under a given set of irradiation conditions. Equation (2) can then be rearranged to determine the D_10_ value (*x*), when *y* = 0.1*a* (i.e. a 90% loss of starting titre):

(3) \begin{equation*} {D}_{10}=\frac{\mathit{\ln}(0.1)}{-b} \end{equation*}

Therefore, the D_10_ is higher where *b* is lower, as would be expected for more radioresistant pathogens. The line of best fit, D_10_ values and DS_SAL_ were determined for IAV and NDV (Table 1), and ZIKV and SFV (Table 2). The D_10_ values of IAV and NDV were comparable (2.1 and 2.8 kGy, respectively), whereas SFV had a higher D_10_ than ZIKV for dry-ice irradiation (5.5 compared to 4.2 kGy). The D_10_ values were also calculated for ice and RT and were comparable, however an exact D_10_ value for RT-irradiated SFV could not be determined since virus was undetectable at the lowest irradiation dose used (5 kGy) in our experimental settings. Importantly, calculating a D_10_ value for pathogens with single-hit kinetics allowed us to calculate the DS_SAL_ using equation (1), as shown in Tables 1 and 2. However, calculating the DS using equation (1) would not be possible for pathogens with multiple-hit kinetics as ignoring the shoulder of resistance would result in a miscalculation of the DS. Therefore, we propose a new formula to calculate the DS_SAL_ that could accommodate both single-hit and multiple-hit inactivation kinetics:

(4) \begin{equation*} {DS}_{SAL}=R+\left(n\times{D}_{10}\right) \end{equation*}

where *R* refers to the irradiation dose required to overcome the shoulder of resistance with a value of ‘*R* = 0’ for pathogens that show linear inactivation curves (single-hit kinetics). This formula takes into account the distinct regions of multiple-hit curves and should allow for more accurate calculation of DSs.

When considering the inactivation curve of dry-ice irradiated RV **(**Fig. 4**),** we could consider 40 kGy to be required to overcome the radioresistance (R value). We could also calculate the D_10_ for the radiation sensitive portion of the curve (above 40 kGy) using equation (3). The D_10_ for the linear portion of the curve was calculated to be 3.2 kGy (based on the formula *y* = 7 × 10^15^ × e^−0.718*x*^). To calculate DS_SAL_ using equation (4), we need to estimate the number of log_10_ reduction in virus titre (*n*) required to achieve the internationally acceptable SAL of 10^−6^. For this calculation, the viable titre at *x =* 40 kGy was determined to be 2.4 × 10^3^ FFU/mL. Thus, a further reduction of 9.4 log_10_ will be required to meet a SAL of 10^−6^. Hence the DS_SAL_ for dry-ice irradiated RV could be calculated based on equation (4) as follows:

DS_SAL_ = 40 + (9.4 × 3.2) = 70.08 kGy.

**Fig. 5.**
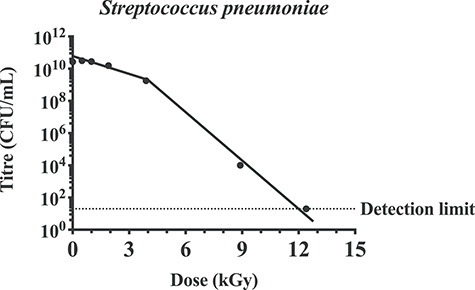
Inactivation curve of *S. pneumoniae* demonstrates multiple-hit kinetics. *Streptococcus pneumoniae* was irradiated on dry-ice (DI) at the indicated doses. Titre was measured by colony forming units and data are presented as mean ± SEM (*n* = 4). Inactivation curve demonstrates a multiple hit kinetics and a shoulder of resistance that require an irradiation dose of 4 kGy.

To confirm the applicability of this method, we considered the inactivation curve of the bacterial pathogen *S. pneumoniae*. This pathogen has a double-stranded genome, and the inactivation curve is non-linear (Fig. 5). The shoulder of resistance, or R value, was determined to be 4 kGy. At *x* = 4 kGy the titre was 1.7 × 10^9^ CFU/mL, thus 15.2 log_10_ reductions (*n* = 15.2) were required to reach the accepted SAL level of 10^−6^. We calculated the D_10_ value for the log–linear curve (after 4 kGy) using the formula *y* = 6 × 10^13^ × *e*^−2.611*x*^, which shows a value of 0.88 kGy. Therefore, the DS_SAL_ for *S. pneumoniae* irradiated on dry-ice could be calculated using equation (4) as follows:

DS_SAL_ = 4 + (15.2 × 0.88) = 17.38 kGy.

## DISCUSSION

Current recommendations for calculating DSs are based on concepts and formulae generated to meet requirements to sterilize food, medical equipment and other health care products [25, 27, 32]. A dose of 25 kGy is considered the ‘gold standard’ [25] and is often substantiated for a low bioburden. In general, the contaminating species are typically bacteria, which are more sensitive to γ-radiation than viruses [24] and spores [33]. In addition, the The International Organization for Standardization (ISO) suggests that a bioburden of 10^6^ infectious units is unusually high [27]. However, materials prepared for biomedical analysis as well as for vaccine purposes are expected to have bioburden levels much higher than 10^6^ infectious units. Consequently, a DS_SAL_ <25 kGy was not observed for any of the viruses irradiated on dry ice (Tables 1 and 2). Accordingly, 25 kGy should not be considered a DS for virally contaminated materials, nor for vaccine inactivation purposes, without properly addressing the inactivation curve and D_10_ value, particularly when frozen materials are irradiated using dry ice. For pathogens that pose a biosecurity concern a dose of 50 kGy is usually considered sufficient [28]. However, a SAL of 10^−6^ could not be reached following irradiation with 50 kGy on dry ice for ZIKV or SFV, or at 50 kGy using all irradiation conditions (dry ice, ice and RT) for RV (Table 2). Therefore, existing concepts that govern the use of γ-irradiation to sterilize highly infectious pathogens should be carefully considered to ensure sterility at internationally accepted levels. This will be essential for the development of highly safe and immunogenic γ-irradiated vaccines.

Inactivation curves typically follow single-hit or multiple-hit kinetics. It was expected that inactivation of single-stranded, non-segmented RNA viruses would follow single-hit kinetics. This was confirmed with NDV (Fig. 2B), SFV and ZIKV (Fig. 3), as well as previous publications [16, 34]. Interestingly, IAV also appeared to follow single-hit inactivation kinetics despite having segmented single-stranded RNA genomes (Fig. 2A). We have previously demonstrated log–linear inactivation of IAV [35]. Previous reports of inactivation curves of viruses with single-stranded segmented genomes have also demonstrated first-order kinetics [17, 36]. Conversely, the inactivation curves of RV (Fig. 4) and *S. pneumoniae* (Fig. 5) demonstrate multiple-hit inactivation kinetics where an accumulation of damage is required to sterilize each pathogen. Unlike other viruses used in this study, the genome of RV is comprised of 11 dsRNA segments and sufficient damage to both strands will be required to completely inactivate any genome segment. In addition, reassortment of RV is relatively frequent, and has been shown to enhance resistance in response to UV treatment [37]. Thus, incomplete inactivation of dsRNA segments accompanied by reassortment can rescue the infectivity of RV. This could explain the large shoulder of 40 kGy observed for RV. In contrast, *S. pneumoniae* cannot reassort, and SOS repair used by other bacterial species such as *Escherichia coli* [38] in response to γ-radiation do not appear to occur in *S. pneumoniae* [39]. However *S. pneumoniae* does utilize some repair mechanisms, such as excision repair [40]. It is also important to consider that *S. pneumoniae* has double-stranded genomes which could enhance resistance as both strands may need to be damaged to ensure inactivation. Conversely, mammalian cells are highly susceptible to γ-radiation despite having double-stranded genomes and repair mechanisms [41, 42]. This is particularly relevant to the development of γ-irradiated cancer vaccines such as GVAX, which is currently in clinical trials [43]. DSs reported are typically between 35 [44] and 100 Gy [45]. The radiosensitivity of mammalian cells is explained by a considerably larger genome than viruses and bacteria.

The ISO recommendations for calculating the DS involves setting a dose based on the calculated bioburden and a standard distribution of resistances (SDR) based on a D_10_ of between 2 and 3 kGy [27]. Where radioresistance is higher than the SDR (as would be the case for most viruses), the preparation is subjected to incremental increases in radiation dose and the proportion of positive samples is used to calculate the DS (i.e. at a SAL of 10^−2^, there should be 0, 1 or 2 positive samples out of 100 for statistically significant substantiation of the dose used). However, extrapolating this data for a SAL of 10^−6^ does not take into account the potential for non-linear inactivation. We have proposed an alternative method where the shoulder of resistance is calculated and accounted for as well as log–linear inactivation. To ensure the sterility and safety of irradiated materials, it is important to take into account the shape of the inactivation curve when considering the SAL, and equation (4) allows the shoulder of resistance to be incorporated when calculating the DS for pathogens that display multiple-hit inactivation kinetics. Importantly, mathematical modelling must also be coupled with rigid sterility testing.

It is important to note that γ-rays cause damage to pathogens by directly interacting with genomes to cause cross-linking, and single- and double-stranded breaks [46–49], and can interact with water or oxygen molecules to form free radicals. Oxidative damage causes most of the protein damage [20], but the formation and movement of free radicals can be reduced in frozen samples [50, 51]. In fact, irradiating frozen prions at incredibly high doses of up to 200 kGy showed minimal loss of transmission [52], demonstrating the resistance of proteins to γ-radiation at low temperatures. Thus, while irradiating at higher temperatures is more effective for sterilization (Figs 3 and 4, [16, 17, 19]), irradiating frozen samples is expected to better maintain structural integrity [35, 53]. Therefore, γ-irradiation has routinely been performed at low temperatures to obtain more effective results for both biomedical analysis and vaccine immunogenicity. However, our data clearly illustrate that sterility at an internationally accepted level based on SAL of 10^−6^ could not be achieved when irradiating high titres of some pathogens with 50 kGy using dry-ice conditions, and even when using room-temperature irradiation for radioresistant pathogens such as RV. Therefore, to ensure the safety of irradiated materials, the irradiation temperature, the appropriate method to calculate DS_SAL_ and rigid sterility testing must be considered. Overall, this study highlighted a serious gap in current practices, and we propose a new mathematical formula to calculate both the D_10_ value and DS_SAL_ to ensure the safety of irradiated materials for vaccine and research purposes.
